# System-level modeling with temperature compensation for a CMOS-MEMS monolithic calorimetric flow sensing SoC

**DOI:** 10.1038/s41378-024-00853-8

**Published:** 2025-01-20

**Authors:** Linze Hong, Ke Xiao, Xiangyu Song, Liwei Lin, Wei Xu

**Affiliations:** 1 State Key Laboratory of Radio Frequency Heterogeneous Integration, Shenzhen University, 518060 Shenzhen, China; 2 College of Electronics and Information Engineering, Shenzhen University, 518060 Shenzhen, China; 3 Department of Mechanical Engineering, University of California, Berkeley, 94720-1740 CA USA

**Keywords:** Electrical and electronic engineering, Physics

## Abstract

We present a system-level model with an on-chip temperature compensation technique for a CMOS-MEMS monolithic calorimetric flow sensing SoC. The model encompasses mechanical, thermal, and electrical domains to facilitate the co-design of a MEMS sensor and CMOS interface circuits on the EDA platform. The compensation strategy is implemented on-chip with a variable temperature difference heating circuit. Results show that the linear programming for the low-temperature drift in the SoC output is characterized by a compensation resistor *R*_c_ with a resistance value of 748.21 Ω and a temperature coefficient of resistance of 3.037 × 10^−3^ °C^−1^ at 25 °C. Experimental validation demonstrates that within an ambient temperature range of 0–50 °C and a flow range of 0–10 m/s, the temperature drift of the sensor is reduced to ±1.6%, as compared to ±8.9% observed in a counterpart with the constant temperature difference circuit. Therefore, this on-chip temperature-compensated CMOS-MEMS flow sensing SoC is promising for low-cost sensing applications such as respiratory monitoring and smart energy-efficient buildings.

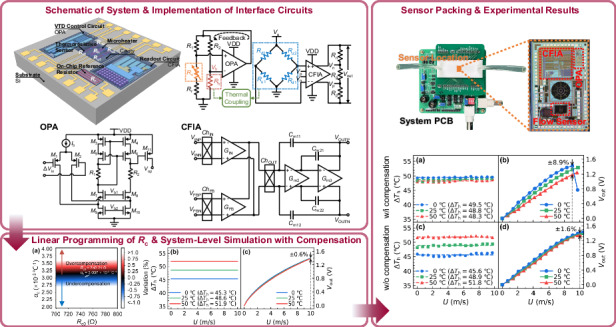

## Introduction

Flow measurement is important for industrial, medical, and biological applications^1,2^, where MEMS thermal flow sensors are suitable for these applications due to their low cost, compact size, and low power consumption^3–6^. In particular, advancements in CMOS-MEMS technology have enabled direct circuit-sensor integration to enhance performance in thermal flow sensing systems. Since there are no movable microstructures in MEMS thermal flow sensors, they can be easily integrated with application-specific integrated circuits (ASICs) in CMOS technology through either monolithic or hybrid integration^7–10^. This integration not only improves the signal-to-noise ratio of the sensing system but also optimizes the chip area utilization, thereby reducing overall development costs^11,12^.

The expanding use of thermal flow sensors, particularly during the COVID-19 pandemic, has highlighted their critical role in respiratory monitoring, where accurate flow measurements are essential^13–16^. However, thermal flow sensing systems often experience signal drifts due to changes in the ambient temperature, which impacts sensor performance across diverse working environments^17–19^. Consequently, temperature compensation is crucial to ensure high-precision thermal flow sensing.

Various flow sensing systems with ambient temperature compensation schemes have been reported, which can be classified into two types: hardware-based circuitry compensation and software-based algorithm compensation. Software-based algorithm compensation schemes typically involve back-end algorithm configurations enabled by programmable resistors on a microcontroller unit (MCU) or semi-empirical models^20,21^. These compensation schemes often require a learning process for each sensor, which is time-consuming with tasks like parameter extraction and sensor calibration^20,21^. Conversely, hardware-based circuitry compensation schemes often utilize temperature sensors^22–28^ with associated Wheatstone bridge circuits to enhance sensor performance^24^. This approach relies on circuit configuration with theoretical analyses, mathematical calculations, and simulations. However, most researchers study the temperature drift issue from the sensor body without considering the interface circuit^21–27^. As such, there is no reported scheme dealing with the temperature drift problem in the hardware setup of an integrated flow sensing system on chip (SoC).

In our earlier work, we have developed a one-dimensional (1-D) model to enhance the efficiency of MEMS design processes^29^. Building on this model, we have subsequently implemented the temperature compensation scheme for the MEMS sensor without considering the influence of the interface circuit^27^. Afterward, we carried out a comprehensive system architecture for a new flow sensing SoC, focusing on the optimization of key metrics such as sensitivity, power consumption, detection limit, and footprint, without addressing temperature compensation^30^. Drawing on these efforts and leveraging the temperature compensation framework proposed in our prior simulation work^28^, this paper further establishes a system-level EDA simulation platform with a linear programming (LP) model for the on-chip temperature compensation resistor with experimental validations, thereby achieving the temperature drift compensation for the CMOS-MEMS flow sensing SoC. This approach produces consistent outputs from the monolithic chip to substantially minimize the sensor calibration or off-chip circuit regulation. Experimental results show that the on-chip temperature-compensated SoC maintains a minimal temperature drift of only ±1.6% within the temperature range of 0–50 °C, which aligns closely with theoretical predictions.

The rest of this article is organized as follows. Section “Theoretical analysis” details the sensor system architecture with circuit implementation and the system-level modeling of the flow sensing SoC is introduced. Section “Results and discussion” proposes the LP model along with the on-chip temperature compensation scheme, as well as measurement results, and comparisons with theoretical insights. Finally, the Section “Conclusion” concludes this article.

## Theoretical analysis

### Concept of the sensor system

**Fig. 1:**
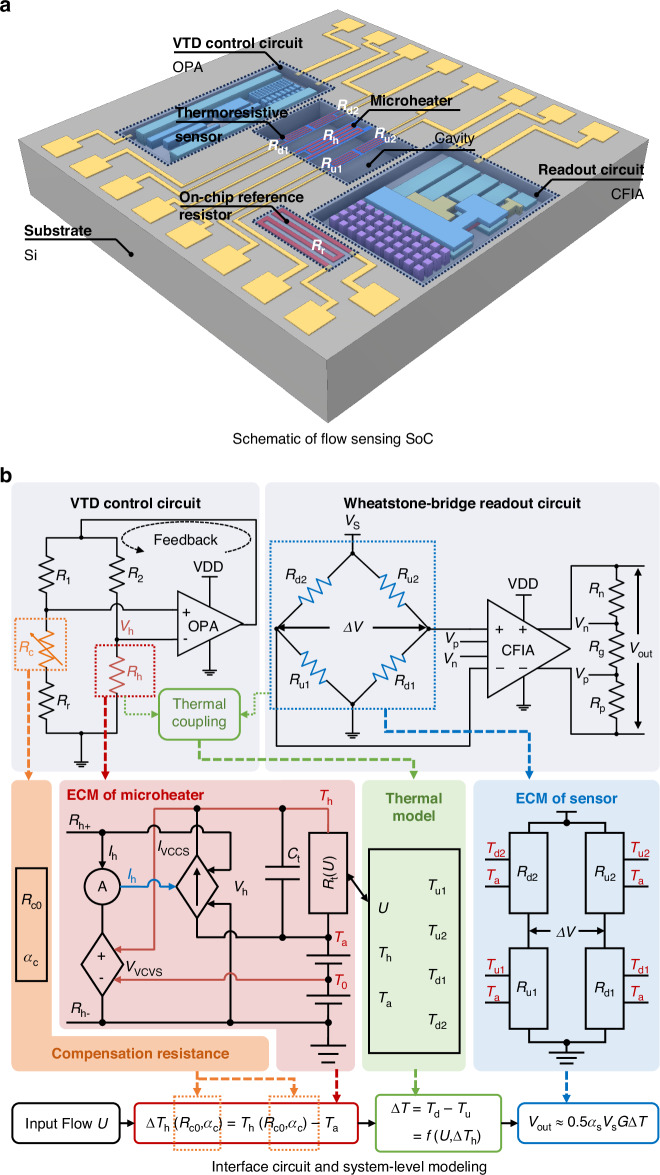
Design and system-level modeling of the CMOS-MEMS thermal flow sensing SoC. **a** Schematic of a monolithically integrated thermal flow sensing SoC based on the 0.18 μm 1P6M CMOS-MEMS technology. **b** System-level model of the flow sensing SoC, where the resistance and TCR of the resistor *R*_c_ will be delicately optimized to achieve on-chip temperature compensation

Figure 1a shows the schematic of a monolithically integrated CMOS-MEMS calorimetric flow sensing SoC. This system comprises dual pairs of symmetrically located upstream and downstream thermistors (*R*_u1_, *R*_u2_, *R*_d1_, and *R*_d2_), and a centrally positioned microheater (*R*_h_), all fabricated from a P+ poly-Si layer. Additionally, it contains a variable temperature difference (VTD) control circuit and a low-noise current feedback instrument amplifier (CFIA) readout circuit, as shown in Fig. 1b. The entire flow sensing SoC is designed and fabricated using the SMIC 0.18 μm 1P6M CMOS technology, while the MEMS sensor structure is subsequently released by an in-house developed post-CMOS process^30^. When a gas flows over the SoC with a speed of *U*, it distorts the temperature distribution around the microheater, resulting in a temperature difference Δ*T* = *T*_d_ − *T*_u_ between the upstream and downstream thermistors. This temperature difference is subsequently converted into a voltage difference via a Wheatstone bridge and amplified by the CFIA with the obtained analog output *V*_out_. Unlike the conventional constant temperature difference (CTD) mode in the previous design^30^, which may cause signal degradation as ambient temperature increases^28^, the VTD control circuit can automatically regulate the overheated temperature Δ*T*_h_ = *T*_h_ – *T*_a_, thereby minimizing the drift of the system output voltage *V*_out_.

### Implementation of interface circuits

**Fig. 2:**
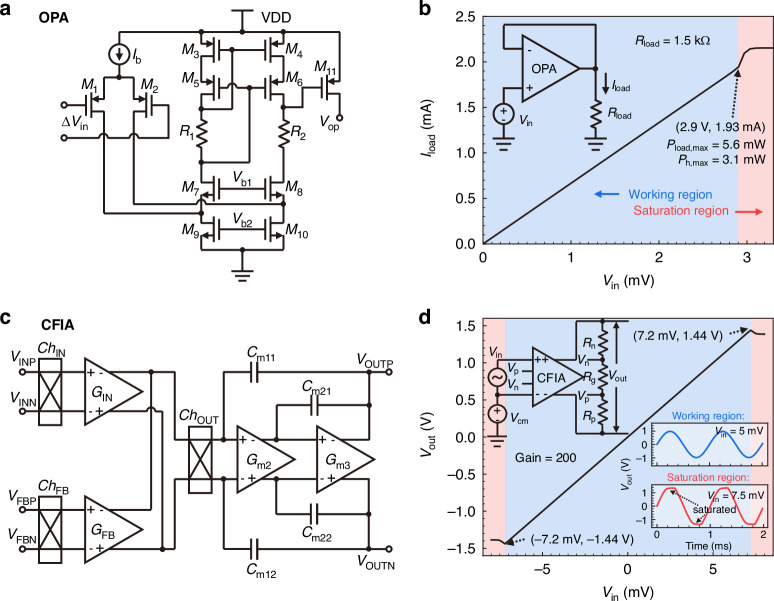
Sensor interface circuit and its performance. **a** Block diagram of the implemented folded-cascode operational amplifier (OPA) with a PMOS buffer for large driving current. **b** The OPA provides an output current drive capability of 1.93 mA for the VTD control circuit with a 1.5 kΩ load, allowing it to deliver a maximum power of 3.1 mW to the microheater, which exceeds the microheater’s maximum required power of 1 mW. **c** Block diagram of the implemented low-noise current feedback instrument amplifier (CFIA). **d** The simulated linear output swing of the proposed CFIA is ±1.44 V, and the pink area indicates that the amplified signal is saturated and distorted

The proposed VTD circuit with a negative feedback loop is shown in the left part of Fig. 1b. In this design, the ratio of resistors *R*_1_/*R*_2_ and (*R*_r_ + *R*_c_)/*R*_h_ in the Wheatstone bridge is configured as *k* = 5:1. To ensure stable and precise feedback, the operational amplifier (OPA, Fig. 2a) is designed with a high gain of 108.6 dB and a phase margin of 62.8°^30^. Meanwhile, as demonstrated by the 1.5 kΩ resistor loading simulation in Fig. 2b, the OPA is designed to deliver a maximum power of 3.1 mW to the microheater. Given that the power needed for the microheater to achieve an overheating of 50 °C is less than 1 mW^30^, the VTD circuit allows the microheater to operate across a wide range of overheated temperature differences Δ*T*_h_ under different flow velocities *U* and ambient temperatures *T*_a_. Specifically, when the whole system is in a balanced state, the overheated temperature difference Δ*T*_h_ of the microheater can be calculated as:

1 $$\Delta {T}_{{\rm{h}}}=\frac{({R}_{{\rm{c0}}}{\alpha }_{{\rm{c}}}+{R}_{{\rm{r0}}}{\alpha }_{{\rm{r}}}-k{R}_{{\rm{h0}}}{\alpha }_{{\rm{h}}})({T}_{{\rm{a}}}-{T}_{0})+({R}_{{\rm{c0}}}+{R}_{{\rm{r0}}}-k{R}_{{\rm{h0}}})}{k{R}_{{\rm{h0}}}{\alpha }_{{\rm{h}}}}$$

where *α*_c_, *α*_h_, and *α*_r_ are the temperature coefficients of resistance (TCRs) of the compensation resistor *R*_c_, the microheater *R*_h_, and the reference resistors *R*_r_, respectively. *R*_c0_, *R*_h0_, and *R*_r0_ represent the resistance values of these components at a reference temperature *T*_0_. In a nominal design (*α*_r_ = *α*_h_), by setting *R*_r0_/*R*_h0_ = *k* and a compensation resistor *R*_c_ with a non-zero TCR, the overheated temperature Δ*T*_h_ can be automatically regulated with the ambient temperature *T*_a_, as shown in Eq. (1). Conversely, by setting *R*_c_ with a zero TCR, the conventional CTD model is achieved with configured *R*_c0_ = $\bar{\alpha }$_r,h_*R*_r0_Δ*T*_h_.

The readout circuit, shown on the right part of Fig. 1b, comprises a Wheatstone bridge formed by two pairs of thermistors and a CFIA (Fig. 2c). This setup is used to amplify mV-level signal output Δ*V* from the Wheatstone bridge. The four thermistors are designed identically, and on account of the symmetry, the resistance values for the upstream and downstream thermistors can each be denoted as *R*_u_ = *R*_u1,2_ and *R*_d_ = *R*_d1,2_, respectively. Meanwhile, the CFIA in the readout circuit is designed with a low noise floor of only 12.4 nV/$\sqrt{{\rm{Hz}}}$^30^, chopped at 20 kHz. In addition, as shown in Fig. 2d, the CFIA is designed with an input signal swing range within ±7.2 mV and a maximum absolute output voltage of 1.44 V (gain = 200). Note that the pink area in Fig. 2d indicates output waveform saturation and distortion when the input signal swing exceeds 7.2 mV. This saturation phenomenon typically occurs at high flow rates, where the MEMS sensor has a high-temperature difference output Δ*T*.

After the circuit amplification, the output of the CMOS-MEMS sensor system can be expressed as:

2 $${V}_{{\rm{out}}}=G\left(\frac{{R}_{{\rm{d}}}}{{R}_{{\rm{u}}}+{R}_{{\rm{d}}}}-\frac{{R}_{{\rm{u}}}}{{R}_{{\rm{u}}}+{R}_{{\rm{d}}}}\right){V}_{{\rm{s}}}\approx 0.5{\alpha }_{{\rm{s}}}{V}_{{\rm{s}}}G\Delta T$$

where *V*_s_ = 1 V is the applied voltage in the Wheatstone bridge, *α*_s_ denotes the TCR of the thermistors *R*_u1,2_ and *R*_d1,2_ at 25 °C, and *G* = (*R*_n_ + *R*_g_ + *R*_p_) / *R*_g_ is the closed-loop gain of CFIA, which is set at 200.

### System-level model of flow sensing SoC

**Fig. 3:**
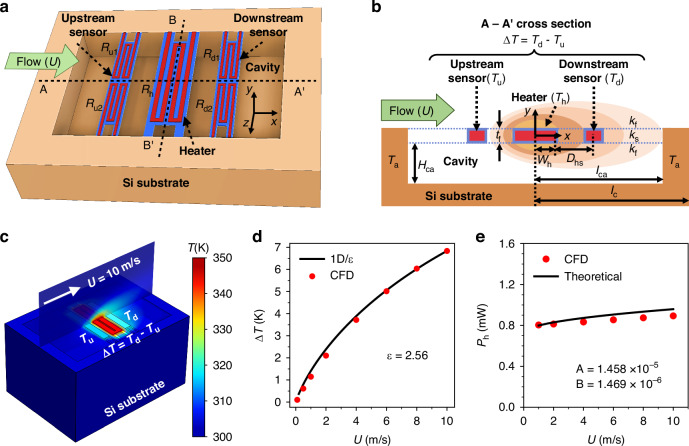
Structural design and performance analysis of the MEMS flow sensor. **a** 3-D structural view of a MEMS calorimetric flow sensor, which consists of a central microheater and two pairs of thermistors arranged symmetrically. **b** A-A′ cross-section of the MEMS sensor with critical structural parameters: 2*l*_c_ = 1500 μm, 2*l*_ca_ = 200 μm, 2*w*_h_ = 31 μm, *D*_hs_ = 13 μm, *H*_ca_ = 50 μm, and *t*_f_ = 2.52 μm. **c** Distorted temperature profile around the microheater at an input gas flow velocity of 10 m/s. **d** Comparison of the sensor’s output between 1-D thermal model and CFD model at the ambient temperature *T*_a_ of 25 °C, and the fitting factor is *ε* = 2.56. **e** Comparison of the microheater power between theoretical and CFD models, where the calculated values are: *A* = 1.458 × 10^−5^ and B = 1.469 × 10^−6^. Note, the overheated temperature of the microheater Δ*T*_h_ is set at 50 °C in these models

For design optimizations, it is essential to establish the equivalent circuit models (ECMs) of the microheater and thermistors. This allows the MEMS structure to be co-designed with the ASIC circuitry, thus facilitating a fully coupled simulation across mechanical, thermal, and electrical domains. To build the ECM of thermistors, it is necessary to determine the temperature distribution surrounding the microheater at a given working temperature when subjected to fluid flows. For this purpose, an analytical thermal model that considers the key heat transfer behavior of the MEMS flow sensor, as shown in Fig. 3a, b, is proposed. Considering that the proposed micro calorimetric flow sensor will be packaged inside a flow channel^29^, the thermal model with the critical design parameters detailed in Fig. 3b can be written as:

3 $${\left[\begin{array}{c}{k}_{{\rm{s}}}{t}_{{\rm{f}}}+\frac{1}{2}{k}_{{\rm{f}}}{\delta }_{{\rm{t}}}+\frac{1}{2}{k}_{{\rm{f}}}{H}_{{\rm{ca}}}\\ -{\rho }_{{\rm{f}}}{c}_{{\rm{f}}}U\left(\frac{{\delta }_{{\rm{t}}}^{2}}{{H}_{{\rm{ch}}}}-\frac{{\delta }_{{\rm{t}}}^{3}}{2{H}_{{\rm{ch}}}^{2}}\right)\\ -\left(\frac{{k}_{{\rm{f}}}}{{\delta }_{{\rm{t}}}}+\frac{{k}_{{\rm{f}}}}{{H}_{{\rm{ca}}}}\right)\end{array}\right]}^{\rm{T}}\left[\begin{array}{c}\frac{{{\rm{d}}}^{2}T(x)}{{\rm{d}}{x}^{2}}\\ \frac{{\rm{d}}T(x)}{{\rm{d}}x}\\ T(x)\end{array}\right]=0$$

where *T*(*x*) is the temperature profile along the *x*-direction; *k*_s_, *k*_f_ represent the thermal conductivity of the thin film and the fluid, respectively; *t*_f_ is the thickness of the thin film; *H*_ca_ and *H*_ch_ represent the height of the MEMS cavity and the flow channel, respectively; *ρ*_f_, *c*_f_, and *U* denote the density, specific heat capacity, and velocity of the fluid; *δ*_t_ is the averaged thermal boundary layer thickness. Note that the values of *T*(*x*), *δ*_t_, *ρ*_f_, *c*_f_, and *k*_f_ are all related to the ambient temperature *T*_a_, and the corresponding formula can be obtained in our prior work^27^.

According to Eq. (3), the analytical solution for the temperature distribution *T*(*x*) of the flow sensing SoC in the streamwise direction (*x*) can be obtained (details of the calculation can be found in the Supplementary Material), allowing for easy calculation of the thermal output Δ*T* between the upstream and downstream thermistors. The computational fluid dynamics (CFD) simulations, shown in Fig. 3c, were conducted to verify the accuracy of the proposed thermal model. As shown in Fig. 3d, both CFD simulations and the 1-D thermal model show a well-matched trend that the thermal output (Δ*T*) increases with the fluid flow (*U*) ranging from 0 m/s to 10 m/s. After validating the proposed thermal model, the calculated upstream and downstream temperatures *T*_u_ and *T*_d_ can be incorporated into the ECM of the thermistors:

4 $${R}_{{\rm{s}}}={R}_{{\rm{s0}}}[1+{\alpha }_{{\rm{s}}}({T}_{{\rm{u}},{\rm{d}}}-{T}_{0})]$$

where *R*_s_ represents the resistance of the upstream thermistors (*R*_u1,2_) or the downstream thermistors (*R*_d1,2_), *R*_s0_ is the resistance at room temperature (*T*_0_ = 25 °C). By implementing the formula Eqs. (3) and (4) in the form of Verilog-A in the EDA platform, the ECM for the analog behavior description of the thermistors (as shown in the blue part of Fig. 1b) can be established.

The establishment of the thermal model in Eq. (3) and its subsequent ECM are based on the assumption of a constant overheated temperature of Δ*T*_h_ for the microheater. To achieve a fully coupled simulation for the microheater and thermistors in the EDA platform, it is necessary to build the ECM of the microheater to accurately predict its heating temperature under the excitation of the ASIC circuit. According to the principle of energy conservation, the power consumption *P*_h_ of the microheater in the transient state is given as:

5 $${P}_{{\rm{h}}}=(A+B\sqrt{U})\Delta {T}_{{\rm{h}}}+{\rho }_{{\rm{h}}}{\nu }_{{\rm{h}}}{c}_{{\rm{h}}}\frac{{\rm{d}}{T}_{{\rm{h}}}}{{\rm{d}}t}$$

where *V*_h_ and *I*_h_ denote the voltage and current of the microheater, *ρ*_h_, *ν*_h_, and *c*_h_ denote the density, volume, and heat capacity of the microheater, and *t* is the time. Besides, *A* is the heat loss that considers the sum of conduction, while *B*$\sqrt{U}$ represents forced convection by the boundary layer flow, with both factors being temperature-dependent. Note that the parameters *A* and *B* can be determined from the analytical thermal model of the microheater described in the Supplementary material. For the microheater reported in this paper, the values of *A* = 1.458 × 10^−^^5^ and *B* = 1.469 × 10^−6^ are calculated at *T*_a_ = 25 °C. Similarly, as shown in Fig. 3e, CFD simulations are used to verify the thermal model of the microheater. Specifically, under an overheated temperature of Δ*T*_h_ = 50 °C, the thermal model predicted that the power of the microheater *P*_h_ increases as the flow *U* increases and shows high consistency with the CFD simulation results. As can be seen from Eqs. (3) to (5), the thermal coupling phenomenon between the microheater and the thermistors can be revealed. However, the complex interactions between the microheater, the control circuit, and the fluid have not been adequately described. Given the absence of a platform to integrate CFD and EDA simulations, we modeled the microheater as an ECM within the EDA platform to capture the interactions among the fluid flow, microheater power, and heating temperature. This facilitated a comprehensive simulation with the VTD control circuit incorporating negative feedback.

As shown in Fig. 1b, to calculate the overheated temperature of the microheater under the real circuit condition, the microheater’s power *P*_h_ is equivalent to a current *I*_VCCS_ using a voltage-controlled current source (VCCS). Meanwhile, the term (*A* + *B*$\sqrt{U}$)^−^^1^ is equivalent to a resistance *R*_t_(*U*), while *ρ*_h_*ν*_h_*c*_h_ is equivalent to a capacitance *C*_t_, and the targeted Δ*T*_h_ is equivalent to the voltage across both the resistance *R*_t_(*U*) and the capacitance *C*_t_. In addition, as the resistance *R*_h_ of the microheater can be expressed by a similar formula in Eq. (4), the voltage across the microheater can be modeled with a voltage-controlled voltage source (VCVS) as *V*_VCCS_ = *V*_h_ = *I*_h_*R*_h_(*T*_h_). Note that all these expressions can be implemented using Verilog-A in the EDA platform, as shown in the red part of Fig. 1b.

## Results and discussion

### Compensation strategy and simulation results

**Fig. 4:**
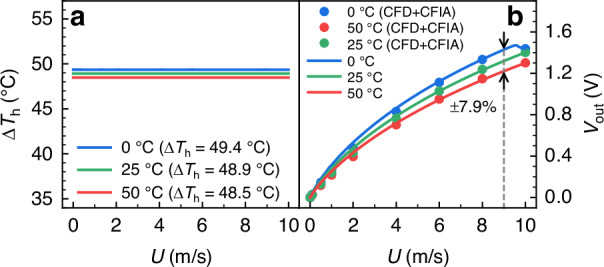
Simulated overheated temperature Δ*T*_h_ and system output *V*_out_ of the flow sensing SoC in the CTD mode under different ambient temperatures. **a** Δ*T*_h_ remains almost constant for the sensor in the CTD mode; **b** *V*_out_ in the CTD mode (±7.9% at 9 m/s). Note that the system-level model is verified using the CFD results with the CFIA readout circuit at 0 °C, 25 °C, and 50 °C

Based on the proposed system-level model, the Spectre simulator in the Cadence Virtuoso platform is used to realize the system-level optimization of the CMOS-MEMS flow sensing SoC. Figure 4 shows the simulated overheated temperature of the microheater in an initially intended CTD mode (by setting *R*_c0_ = 750 Ω, *α*_c_ = 0), along with the signal variation of the SoC output under ambient temperatures ranging from 0 °C to 50 °C. Simulation results were initially validated using CFD results at 0 °C, 25 °C, and 50 °C, combined with the CFIA readout circuit, to confirm the accuracy of the system-level model. Note that these simulations utilize the experimentally determined resistance and TCR values for *R*_h_, *R*_r_, and four thermistors. In our current flow sensing SoC, detailed experimental measurements indicate that they exhibit similar TCR, measuring *R*_h0_ = 1018.9 Ω, *α*_h_ = 2.944 × 10^−^^3^ °C^−1^, and *R*_r0_ = 5104.3 Ω, *α*_r_ = 2.830 × 10^−3^ °C^−1^ at 25 °C, respectively. For the four thermistors, the average *R*_s0_ = 4055.7 Ω and *α*_s_ = 2.897 × 10^−3^ °C^−1^ are used.

As shown in Fig. 4a, the EDA simulation results with the integrated transistor-level ASIC circuit, indicate that the microheater can operate in a CTD mode of Δ*T*_h_ = 50 °C. Additionally, due to the strong driving capability of the interface circuit, changes in the flow rate have a minimal impact on Δ*T*_h_ (<0.01 °C). However, as shown in Fig. 4b, the flow sensing SoC output in the CTD mode decreases as the ambient temperature increases, showing a ± 7.9% change (refer to SoC output at 25 °C) at 9 m/s. Note that due to the limited output swing of the CFIA, the simulated sensor output at *T*_a_ = 0 °C is saturated for a gas flow velocity larger than 9 m/s.

**Fig. 5:**
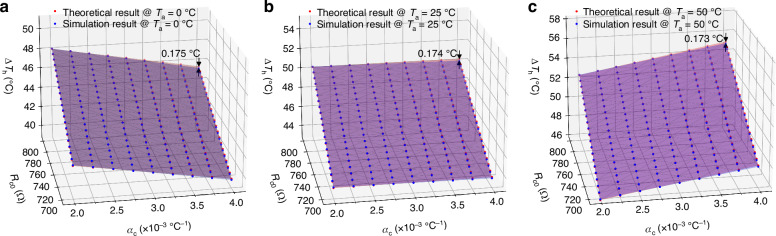
The determined Δ*T*_h_ of the microheater by Eq. (1) and compared to system-level model results. **a** *T*_a_ = 0 °C, **b** *T*_a_ = 25 °C, **c** *T*_a_ = 50 °C. Note that the maximum error between the mathematical formula and the system-level model is less than 0.175 °C

To address the observed negative temperature drift of the integrated sensor system chip operating in the CTD mode, an improvement to the variable temperature difference (VTD) mode with automatically regulated heating temperature is proposed by setting *R*_c_ with a positive temperature coefficient (PTC). To configure and verify the VTD functionality for temperature compensation, the resistance value of *R*_c0_ is initially selected from a range of 700–800 Ω, while *α*_c_ is determined within the common TCR values of PTC resistors in the 0.18 μm CMOS process, which ranges from 2 × 10^−^^3^ to 4 × 10^−3^ °C^−1^. As shown in Fig. 5, by comparing the simulation results of the system-level model with the ideal results calculated by Eq. (1), the overheated temperature of the microheater can be easily regulated from 40 °C to 56 °C by the configured VTD circuit, with a maximum deviation from the ideal case of less than 0.175 °C.

To further assess the impact of ambient temperature variations on the system output in the VTD mode, the targeted temperature drift value used for linear programming (LP) optimization is defined as:

6 $${{Variation}}=\frac{{V}_{{\rm{out}}}({T}_{{\rm{a}}}=50\,^{\circ} {\rm{C}})-{V}_{{\rm{out}}}({T}_{{\rm{a}}}=0\,^{\circ} {\rm{C}})}{{V}_{{\rm{out}}}({T}_{{\rm{a}}}=0\,^{\circ} {\rm{C}})+{V}_{{\rm{out}}}({T}_{{\rm{a}}}=50\,^{\circ} {\rm{C}})}$$

**Fig. 6:**
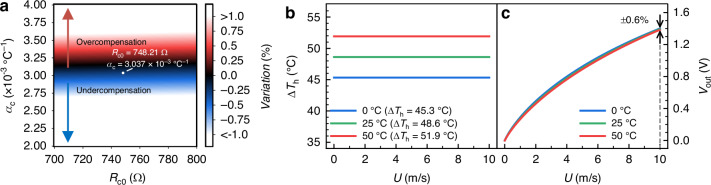
Optimization and simulation of temperature-compensated interface circuit. **a** Linear programming (LP) range of the resistor *R*_c_ based on the on-chip temperature compensation of the system output, where red indicates overcompensation, and blue indicates undercompensation. Note that the compensation resistor is set at a resistance of *R*_c0_ = 748.21 Ω with a TCR of *α*_c_ = 3.037 × 10^−3^ °C^−1^ at 25 °C. **b**, **c** Simulated overheated temperature Δ*T*_h_ and system output *V*_out_ of the flow sensing SoC in the VTD mode under different ambient temperatures: **b** Δ*T*_h_ of the microheater in the VTD mode (Δ*T*_h_ with PTC); **c** *V*_out_ in the VTD mode (±0.6% at 10 m/s)

According to Eqs. (1), (2), (3), and (6), an LP model between *Variation* and the parameters *R*_c0_ and *α*_c_ of the temperature compensation resistor can be formulated as follows:

7 $$\begin{array}{c}\begin{array}{cc}\min & |{{Variation}}|+{\varepsilon }_{\mathrm{var}}\end{array}\\ \begin{array}{cc}{\rm{s}}{\rm{.t}}. & 700\,\Omega \,\le \,{R}_{{\rm{c0}}}\,\le \,800\,\Omega \end{array}\\ 2\,\times \,{10}^{-3}\,{\scriptstyle{\circ}\atop} {{\rm{C}}}^{-1}\,\le \,{\alpha }_{{\rm{c}}}\,\le \,4\,\times \,{10}^{-3}\,{\scriptstyle{\circ}\atop} {{\rm{C}}}^{-1}\end{array}$$

where *ε*_var_ is the design tolerance set to facilitate the selection of the temperature compensation resistor, and the maximum value of *ε*_var_ is 1%. As shown in Fig. 6a, the colored region represents the selection range of *R*_c_ and *α*_c_ that satisfies a temperature drifting less than ±1%, where the red portion indicates overcompensation (i.e., *Variation* > 0), and the blue portion represents undercompensation (i.e., *Variation* < 0). To configure a compensation resistor in the colored region of Fig. 6a, we parallel-connected a PT100 resistor with a PT500 resistor, then series-connected this combination with another set of PT100 and PT500 resistors. This configuration forms the *R*_c_ with a resistance value *R*_c0_ of 748.21 Ω and TCR of 3.037 × 10^−3^ °C^−1^ at 25 °C, which can be readily implemented using an N+ diffusion resistor with silicide in the common 0.18 μm CMOS process and monolithically integrated within the SoC.

Figure 6b, c shows the simulated Δ*T*_h_ and SoC output under the VTD mode from the system-level model after applying the resistance value and TCR of *R*_c_ into the circuit system. The entire sensing system is evaluated with an input flow range of 0–10 m/s and an ambient temperature range of 0–50 °C. It is observed that the overheated temperature of the microheater under the VTD mode is automatically regulated, and the temperature drift of the sensor output is reduced to ±0.6% at 10 m/s in contrast to the non-compensated counterpart shown in Fig. 4.

### Experimental results

**Fig. 7:**
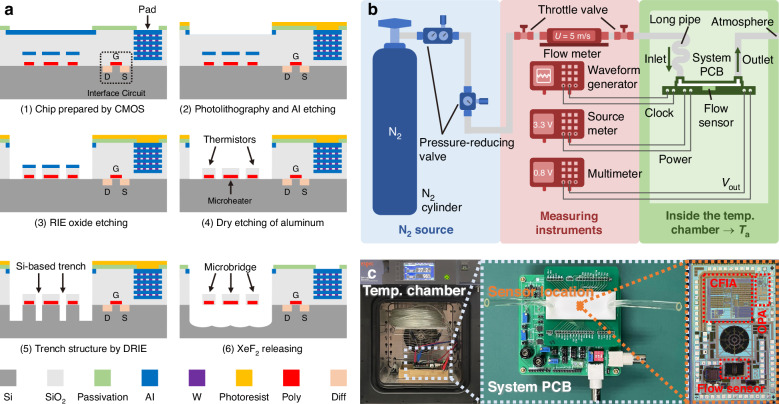
Fabrication and experimental setup of the flow sensing SoC. **a** Fabrication process of the proposed calorimetric flow sensing SoC. **b** Experimental setup for the calorimetric flow sensing SoC with nitrogen gas flow. **c** Experimental configuration in the temperature chamber, system-packaged PCB with a 3D printed flow channel, and microscopic image of the fabricated monolithic calorimetric flow sensing SoC

**Fig. 8:**
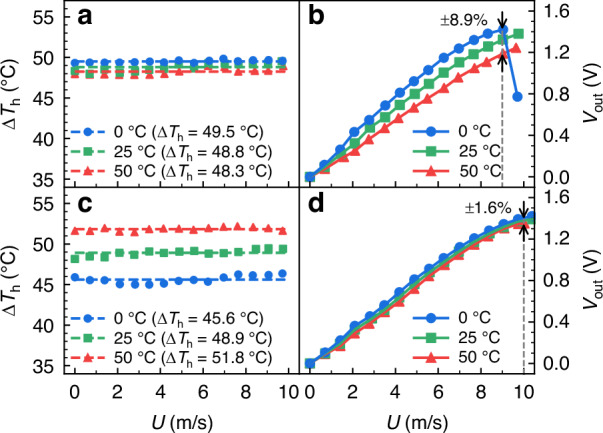
Comparison of the overheated temperature Δ*T*_h_ and system output *V*_out_ of the flow sensing SoC in CTD and VTD modes under different ambient temperatures. **a** Δ*T*_h_ remains almost constant for the sensor in the CTD mode; **b** *V*_out_ in the CTD mode (±8.9% at 9 m/s); **c** Δ*T*_h_ of the microheater in the VTD mode (Δ*T*_h_ with PTC); **d** *V*_out_ in the VTD mode (±1.6% at 10 m/s)

The sensor used to verify the on-chip temperature drift strategy is fabricated using an in-house designed post-CMOS process (Fig. 7a) and subsequently tested in a temperature chamber with nitrogen flow (Fig. 7b, c). The measured overheated temperature and system output of the flow sensing SoC before and after temperature compensation is shown in Fig. 8. Both the compensated and non-compensated experimental results indicate similar output trends to the system-level simulation results. It should be noted that due to the resolution (6.5-digit) of the multimeter (DMM6500, Tektronix, USA), there will be a slight fluctuation error in the calculated Δ*T*_h_ based on the feedback voltage and the microheater’s voltage of the VTD control circuit^27^. For the final output of the sensing SoC, within the ambient temperature range of 0–50 °C, the determined temperature drift of the non-compensated sensor system is ±8.9% at 9 m/s, while it is reduced to ±1.6% at 10 m/s after on-chip compensation. Similar to the system-level simulation results in Fig. 4b, an early saturation in high-speed flow is observed in the sensor output for the non-compensated system under 0 °C, due to the limited swing range of CFIA, as shown in Fig. 8b. However, as shown in Fig. 8d, after applying the temperature compensation scheme, both the 0 °C and 50 °C curves align with the 25 °C curve to mitigate the saturation issue in the 9–10 m/s flow range. Additionally, due to the variations in the microfabrication process, the resistance values and TCR of four thermistors are not identical at 25 °C. These fabrication variations make it impractical for the simulation to account for specific resistance parameters in each chip. Conversely, the utilization of the matched resistance *R*_s0_ and TCR *α*_s_ for four thermistors at 25 °C, results in an ~1% discrepancy between simulation and experimental results.

Table 1 summarizes the performance of the proposed temperature-compensated flow sensing system and compares it with the state-of-art. Notably, few reported temperature-compensated flow sensing systems feature CMOS-MEMS monolithic integration; and some of these sensing systems with MEMS technology were fabricated using materials incompatible with CMOS processes^26^. In contrast, our approach utilizes the CMOS-MEMS fabrication combined with a proprietary in-house developed post-CMOS process. This represents a significant improvement over our previous work^27^, in which only the MEMS sensor is integrated via the CMOS process, while the remaining circuitry is implemented on the PCB. In comparison, the current work incorporates on-chip circuitry for temperature compensation, eliminating the need for algorithmic parameter extraction or calibration, thereby substantially improving design efficiency and resource utilization. Remarkably, the proposed system-level simulation model accurately predicts the on-chip temperature compensation effects, thus eliminating the need for multiple experimental iterations. Furthermore, such a model in EDA also broadens the scope to consider the overall impact of temperature on the entire sensing system, including both the MEMS sensor and the interface circuit. Experimental validation affirms the feasibility of the proposed system-level model and on-chip temperature compensation technique, demonstrating a leading performance among the state-of-the-art works with a ± 1.6% temperature drift within the ambient temperature range of 0–50 °C.

**Table 1:** Performance comparison with state-of-the-art temperature-compensated thermal flow sensing systems

Ref.	System packaging	Power consumption (mW)	Sensitivity	Response time (ms)	Flow range (m/s)	Temperature drift	Compensation type
^21^	MEMS & PCB circuit	N/A	N/A	N/A	0–20; N/A^LR^	±3.5%^Sen^ (−20 to 40 °C)	Software-based algorithm compensation
^26^	MEMS & PCB circuit	N/A	N/A	36^b^	0–6.37^a^; N/A^LR^	2.5%^Sen^ (20–34 °C)	Hardware-based circuitry compensation
^27^	CMOS-MEMS & PCB circuit	<2.77^PH^	543 mV/(m/s)	N/A	−11 to 11; −1.3 to 1.3^LR^	0.5%^Sen^ (22–48 °C)	Hardware-based circuitry compensation
^20^	MEMS & PCB circuit	<158.1^PH^; <333.5^PS, a^	N/A	N/A	0–33; N/A^LR^	2.7%^Sys^ (0–40 °C)	Software-based algorithm compensation
This work	CMOS-MEMS monolithic	<0.92^PH^; <4.96^PS^	156 mV/(m/s)	87.5^b^	−10 to 10; −6 to 6^LR^	±1.6%^Sys^ (0–50 °C)	Hardware-based circuitry compensation

N/A = Specification not available

^a^Estimated from available data

^b^The time taken for a sensor to reach 90% of the final value

^Sen^Sensor-level compensation

^Sys^System-level compensation

^LR^Linear range of MEMS thermal flow sensors

^PH^Heating power

^PS^System power

## Conclusion

This paper presented a system-level simulation model on the EDA platform and an on-chip hardware circuitry temperature compensation technique for the optimization of a monolithic CMOS-MEMS calorimetric flow sensing SoC. The approach involved modeling the microheater and thermistors of the flow sensor as ECMs, and constructing a multi-physics system-level simulation model encompassing mechanical, thermal, and electrical domains. Subsequently, an LP model was employed to identify the parameter selection range for the critical compensation resistor. The effectiveness of the temperature compensation was evaluated through system-level simulations and experimental validations. Ultimately, we successfully minimized the system’s temperature drift to ±1.6% within a tested temperature range of 0–50 °C and enabled the bi-directional flow sensing in a range of −10 to 10 m/s. The development of this high-performance calorimetric flow sensing SoC holds significant potential for societal and economic benefits by addressing precise flow monitoring needs in industries, the Internet of Things (IoT), and healthcare applications.

## Materials and methods

### Fabrication process

The calorimetric flow sensing SoC requires a post-CMOS process to release the MEMS structure as shown in Fig. 7a. Initially, the CMOS-fabricated sensor chip undergoes photolithography, revealing the top metal layer (M6) in the sensor’s suspended region. Subsequently, dry etching is employed to remove the exposed top metal layer, followed by reactive ion etching (RIE) to etch the silicon dioxide down to the silicon substrate. Similarly, dry etching is used to remove the protective metal (M2) of the sensor bridge. Deep reactive ion etching (DRIE) is then performed on the silicon substrate to create vertical trenches. Finally, isotropic etching with XeF_2_ is carried out to suspend the sensor structure.

### Experimental setup

The experimental setup is shown in Fig. 7b, c. The fabricated SoC is packaged in a 3-D printed flow channel with a size of 65 mm × 12 mm × 2.5 mm (Length × Width × Height) and embedded in a printed circuit board (PCB). Nitrogen gas (N_2_) flow is released from a N_2_ cylinder and passes through two-stage pressure-reducing valves and a single-stage throttle valve to reach the reference flow meter (AWM5104VN, Honeywell, USA). Throughout the experiment, the SoC with the flow channel and the system PCB are located inside a temperature chamber (SH-222, ESPEC, Japan) to obtain a stable ambient condition. Furthermore, to ensure that the temperature of the N_2_ gas flow is consistent with the ambient temperature set in the chamber, the N_2_ gas will pass through a long pipe within the chamber before entering the 3-D printed flow channel.

## Supplementary information

Supplementary Information
