# Supplementary material for: System-level modeling with temperature compensation for a CMOS-MEMS monolithic calorimetric flow sensing SoC
Source: Microsyst Nanoeng. 2025 Jan 20;11:13. doi: 10.1038/s41378-024-00853-8 (PMC11743593; doi:10.1038/s41378-024-00853-8)
Supplement: Supplementary file 1 — Supplementary Information [file 41378_2024_853_MOESM1_ESM.docx]

## **System-Level Modeling with Temperature Compensation for a CMOS-MEMS Monolithic Calorimetric Flow Sensing SoC**

## Authors

Linze Hong^1,2^, Ke Xiao^1,2^, Xiangyu Song^1,2^, Liwei Lin^3^, and Wei Xu^1,2,*^

## Affiliations

^1^ State Key Laboratory of Radio Frequency Heterogeneous Integration, Shenzhen University, 518060, Shenzhen, China

^2^ College of Electronics and Information Engineering, Shenzhen University, 518060, Shenzhen, China

^3^ Department of Mechanical Engineering, University of California, CA 94720-1740, Berkeley, USA

^*^ Corresponding author: Wei Xu (weixu@szu.edu.cn)

Figure S1: (a) 3-D structural view of a MEMS calorimetric flow sensor, which consists of a central microheater and two pairs of thermistors arranged symmetrically on both sides. (b) A–A' cross-section of the calorimetric flow sensor with key design parameters. (c) Energy conservation of a lumped element (Δ*x*) (excluding the microheater region) for the film temperature calculation in *x*-direction. (d) Energy conservation for the microheater. (e-f) Top view and B–B' cross-section of the central microheater with critical structural parameters.

## Supplementary Section A: Temperature Profile of the Thermistors

As shown in Fig. S1(a) and (b), the simplified thermal model only considers the key heat transfer behavior of the flow sensor in the *x*–*y* plane, and its critical design parameters are also detailed. By further assuming the temperature profile in the *y*-direction to be piecewise linear, the originally required 3-D heat transfer analysis can be reduced to a simple 1-D one, i.e., only in the streetwise (*x*) direction. As shown in Fig. S1(c), considering the energy conservation within the control volume (Δ*x*) (excluding the microheater region), the governing equation for the film temperature calculation in x-direction can be given as:

$$Q_{\text{f},+x}^{\text{conv}}+Q_{\text{f},+x}^{\text{cond}}+Q_{\text{s},+x}^{\text{cond}}=Q_{\text{f},+y}^{\text{cond}}+Q_{\text{f},-y}^{\text{cond}}$$

where *Q* is the thermal energy; the superscripts “conv” and “cond”, represent heat convection and heat conduction, respectively; the subscripts, “s” and “f”, represent the thin film and the fluid, respectively. Upon performing a more detailed analysis on the heat flux across all surfaces of the lumped element Δ*x* (ref. S1) in Fig. S1(c), the governing equation in (1) for the proposed micro calorimetric flow sensor packaged inside a channel^S2^, can be explained as:

$$\left[\begin{matrix} k_{\text{s}}t_{\text{f}}+\frac{1}{2}k_{\text{f}}\delta_{\text{t}}+\frac{1}{2}k_{\text{f}}H \\ -\rho_{\text{f}}c_{\text{f}}U\left(\frac{\delta_{\text{t}}^{2}}{H_{\text{ch}}}-\frac{\delta_{\text{t}}^{3}}{2H_{\text{ch}}^{2}}\right) \\ -\left(\frac{k_{\text{f}}}{\delta_{\text{t}}}+\frac{k_{\text{f}}}{H_{\text{ca}}}\right) \end{matrix}\right]^{T}\left[\begin{matrix} \frac{\text{d}^{2}T(x)}{\text{d}x^{2}} \\ \frac{\text{d}T(x)}{\text{d}x} \\ T(x) \end{matrix}\right]=0$$

where *T*(*x*) is the temperature profile along the *x*-direction; *k*_s_, *k*_f_ represent the thermal conductivity of the thin film and the fluid, respectively; *t*_f_ is the thickness of the thin film; *H*_ca_ and *H*_ch_ represent the height of the cavity and the channel, respectively; *ρ*_f_ , *c*_f_ , and *U* denote the density, specific heat capacity, and velocity of the fluid; *δ*_t_ is the averaged thermal boundary layer thickness, which can be found elsewhere (ref. S2). Note that the values of *T*(*x*), *δ*_t_, *ρ*_f_ , *c*_f_ , and *k*_f_ are all related to the ambient temperature *T*_a_.

Taking thermal boundary conditions of *T*(-*l*_ca_) = *T*(*l*_ca_) = 0 and *T*(-*w*_h_) = *T*(*w*_h_) = Δ*T*_h_, the temperature profile solution of (2) is given as:

$$T(x)=\left\{\begin{matrix} \frac{\Delta T_{\text{h}}\left[\text{e}^{r_{2}x}-\text{e}^{-r_{2}l_{\text{ca}}+r_{1}\left(x+l_{\text{ca}}\right)}\right]}{\text{e}^{-r_{2}w_{\text{h}}}-\text{e}^{-r_{2}l_{\text{ca}}-r_{1}\left(w_{\text{h}}-l_{\text{ca}}\right)}},x\in[-l_{\text{ca}},-w_{\text{h}}) \\ \Delta T_{\text{h}},x\in[-w_{\text{h}},w_{\text{h}}] \\ \frac{\Delta T_{\text{h}}\left[\text{e}^{r_{2}x}-\text{e}^{r_{2}l_{\text{ca}}+r_{1}\left(x-l_{\text{ca}}\right)}\right]}{\text{e}^{r_{2}w_{\text{h}}}-\text{e}^{r_{2}l_{\text{ca}}+r_{1}\left(w_{\text{h}}-l_{\text{ca}}\right)}},x\in(w_{\text{h}},l_{\text{ca}}] \end{matrix}\right.$$

where 2*w*_h_ is the width of the microheater, 2*l*_ca_ is the length of the cavity in *x*-axis, $r_{1,2}=\left( \mathcal{-B\pm}\sqrt{\mathcal{B}^{2}-4\mathcal{AC}} \right)/(2\mathcal{A)}$ are the eigenvalues of linearized governing equation (2), and $\mathcal{[A,B,C]}$ is the coefficient vector in the left part of (2). Therefore, the temperature difference Δ*T* between the upstream and downstream thermistors can be expressed as:

$$\Delta T=T_{\text{d}}-T_{\text{u}}=T(w_{\text{h}}+D_{\text{hs}})-T(-w_{\text{h}}-D_{\text{hs}})$$

where *D*_hs_ is the distance between the microheater and the thermistors in the *x*-direction.

## Supplementary Section B: Power of the Microheater

In the aforementioned Supplementary Section A, the establishment of the thermal model and its subsequent ECM are based on the assumption of a constant overheated temperature of Δ*T*_h_ for the microheater. To achieve fully coupled simulation for the microheater and thermistors in the EDA platform, it is necessary to build the ECM of the microheater to simulate its heating temperature. Considering the heat exchange of the microheater to its surroundings shown in Fig. S1(d), the energy conservation of microheater can be described as:

$$P_{\text{h}}=P_{\text{s},x}^{\text{cond}}+P_{\text{f},-y}^{\text{cond}}+P_{\text{f},x}^{\text{conv}}+P_{\text{s1},-z}^{\text{cond}}+P_{\text{s2},+z}^{\text{cond}}+P_{\text{f},z}^{\text{cond}}$$

where the heat conduction terms from the supporting beams and the surrounding gas in (5) are given by:

$$\left\{\begin{matrix} P_{\text{s},x}^{\text{cond}}=\frac{2k_{\text{s}}L_{\text{h}}t_{\text{f}}}{l_{\text{ca}}-w_{\text{h}}}\Delta T_{\text{h}} \\ P_{\text{f},-y}^{\text{cond}}=\frac{2k_{\text{f}}L_{\text{h}}w_{\text{h}}}{H_{\text{ca}}}\Delta T_{\text{h}} \\ P_{\text{s1},-z}^{\text{cond}}+P_{\text{s2},+z}^{\text{cond}}=\left(\frac{2k_{\text{s1}}W_{\text{b}}t_{\text{f}}}{L_{\text{b}}}+\frac{2k_{\text{s2}}W_{\text{b}}t_{\text{f}}}{L_{\text{b}}}\right)\Delta T_{\text{h}} \\ P_{\text{f},z}^{\text{cond}}=\frac{4k_{\text{f}}(w_{\text{h}}-W_{\text{b}})t_{\text{f}}}{L_{\text{b}}}\Delta T_{\text{h}} \end{matrix}\right.$$

where *L*_h_ is the length of the microheater; *k*_s1_ and *k*_s2_ are the thermal conductivity of the front and back supporting beams, respectively; *W*_b_ and *L*_b_ are the width and length of the supporting beams, respectively. Note that, due to the metal wires for circuit connection passing through the frontend supporting beams, the thermal conductivity of the front (*k*_s1_) and back (*k*_s2_) supporting beams are different.

In addition, the heat convection term in the *x*-direction is given by:

$$P_{\text{f},x}^{\text{conv}}=2\bar{h}(U)w_{\text{h}}L_{\text{h}}\Delta T_{\text{h}}$$

where $\bar{h}(U)$ is the average heat convection coefficient. Here for a constantly heated film, its formula can be given as^S1^:

$$\left\{\begin{matrix} \bar{h}(U)=\frac{\left(l_{\text{c}}+w_{\text{h}}\right)h_{\text{l}}(U)}{w_{\text{h}}}\left[1-\left(\frac{l_{\text{c}}-w_{\text{h}}}{l_{\text{c}}+w_{\text{h}}}\right)^{\frac{3}{4}}\right] \\ h_{\text{l}}(U)=0.332k_{\text{f}}\left[\frac{\mu_{\text{f}}c_{\text{f}}}{k_{\text{f}}}\right]^{\frac{1}{4}}\left[\frac{\rho_{\text{f}}U}{\mu_{\text{f}}\left(l_{\text{c}}+w_{\text{h}}\right)}\right]^{\frac{1}{2}}\left[1-\left(\frac{l_{\text{c}}-w_{\text{h}}}{l_{\text{c}}+w_{\text{h}}}\right)^{\frac{3}{4}}\right]^{-\frac{1}{3}} \end{matrix}\right.$$

where 2*l*_c_ is the length of the chip in *x*-axis, and *μ*_f_ is the dynamic viscosity. Note that *μ*_f_ is also related to the ambient temperature *T*_a_.

Considering the transient power of the microheater and the principle of energy conservation, the total heating power *P*_h_ in (5) can be further calculated as:

$$P_{\text{h}}=(A+B\sqrt{U})\Delta T_{\text{h}}+\rho_{\text{h}}\nu_{\text{h}}c_{\text{h}}\frac{\text{d}T_{\text{h}}}{\text{d}t}$$

where *ρ*_h_, *ν*_h_ and *c*_h_ denote the density, volume and heat capacity of the microheater, and *t* is the time. Besides, *A* is the heat loss that considers the sum of conduction in (5), while $B\sqrt{U}$ represents forced convection by the boundary layer flow in (5). Note that *A* and *B* are related to the ambient temperature *T*_a_, and can be calculated as per (5) and (9).

## References

1. Holman, J. P. Heat transfer 10th edn, Ch. 5 (McGraw Hill, 2010).
2. Xu, W. et al. Theoretical and experimental investigations of thermoresistive micro calorimetric flow sensors fabricated by CMOS MEMS technology. *J. Microelectromech. Syst.* **25**, 954–962 (2016).
